# Electronic Adherence Monitoring in a High-Utilizing Pediatric Asthma Cohort: A Feasibility Study

**DOI:** 10.2196/resprot.5362

**Published:** 2016-06-22

**Authors:** Chén Collin Kenyon, Joyce Chang, Sheri-Ann Wynter, Jessica C Fowler, Jin Long, Tyra C Bryant-Stephens

**Affiliations:** ^1^ Center for Pediatric Clinical Effectiveness and PolicyLab Department of Pediatrics The Children's Hospital of Philadelphia Philadelphia, PA United States; ^2^ Department of Pediatrics The Children's Hospital of Philadelphia Philadelphia, PA United States; ^3^ Department of Pediatrics New York University School of Medicine New York, NY United States

**Keywords:** electronic medication monitoring, adherence, beta-agonists, inhaled steroids, motivational interviewing, community health workers

## Abstract

**Background:**

Inner-city, minority children with asthma have the highest rates of morbidity and death from asthma and the lowest rates of asthma controller medication adherence. Some recent electronic medication monitoring interventions demonstrated dramatic improvements in adherence in lower-risk populations. The feasibility and acceptability of such an intervention in the highest-risk children with asthma has not been studied.

**Objective:**

Our objective was to assess the feasibility and acceptability of a community health worker-delivered electronic adherence monitoring intervention among the highest utilizers of acute asthma care in an inner-city practice.

**Methods:**

This was a prospective cohort pilot study targeting children with the highest frequency of asthma-related emergency department and hospital care within a local managed care Medicaid plan. The 3-month intervention included motivational interviewing, electronic monitoring of controller and rescue inhaler use, and outreach by a community health worker for predefined medication alerts. We measured acceptability by using a modified technology acceptability model and changes in asthma control using the Asthma Control Test (ACT). Given prominent feasibility issues, we describe qualitative patterns of medication use at baseline only.

**Results:**

We enrolled 14 non-Hispanic black children with a median age of 3.5 years. Participants averaged 7.8 emergency or hospital visits in the year preceding enrollment. We observed three distinct patterns of baseline controller use: 4 patients demonstrated sustained use, 5 patients had periodic use, and 5 patients lapsed within 2 weeks. All participants initiated use of the electronic devices; however, no modem signal was transmitted for 5 or the 14 participants after a mean of 45 days. Of the 9 (64% of total) caregivers who completed the final study visit, all viewed the electronic monitoring device favorably and would recommend it to friends, and 5 (56%) believed that the device helped to improve asthma control. ACT scores improved by a mean of 2.7 points (*P*=.05) over the 3-month intervention.

**Conclusions:**

High-utilizer, minority families who completed a community health worker-delivered electronic adherence intervention found it generally acceptable. Prominent feasibility concerns, however, such as recruitment, data transmission failure, and lost devices, should be carefully considered when designing interventions in this setting.

## Introduction

Inner-city children with asthma experience high morbidity and mortality from persistent asthma [1-3]. Compared with their white counterparts, non-Hispanic black children have four times the rate of asthma-related emergency department visits, three times the rate of hospitalization, and five times the mortality. These disparities in adverse asthma outcomes have persisted over time despite advances in treatment [4].

Inhaled corticosteroids (ICS) remain the mainstay of therapy for persistent asthma [5]. However, suboptimal adherence to ICS regimens is a major barrier to achieving asthma control [6]. Self-reported adherence is unreliable and consistently overestimates medication use [7,8]. Actual ICS adherence rates measured using electronic monitoring in clinical studies are low for both adults and children [9-11]. Poor adherence to controller regimens is associated with poor asthma control [12], increased utilization of emergency and hospital care [13,14], and higher mortality from asthma [15,16]. Urban minority youth in particular demonstrate some of the lowest rates of adherence, ranging from 11% to 45% of prescribed doses [11,17-19]. Better strategies to improve adherence are needed to engage this patient population, especially for those experiencing high levels of asthma morbidity. In this setting, interventions involving community health workers have demonstrated great promise [20-22].

Electronic monitoring is a strategy used in a variety of settings to measure and increase medication adherence among patients with chronic conditions [23-26]. Studies in adults with asthma have shown that electronic monitoring interventions can increase adherence to ICS regimens [27-29]. In the few pilot studies of electronic monitoring interventions done in children, behavior modification strategies that included direct feedback about electronically monitored adherence were thought to be critical components of the strategies’ success [30-34]. Motivational interviewing is a behavior modification strategy that has also been shown to improve medication adherence [35,36], and it helps address barriers to adherence such as parental beliefs about medications [37]. Thus far, electronic monitoring studies have not focused on the highest utilizers of emergency and hospital care among inner-city, minority children with asthma. By targeting this highest-risk group, an electronic adherence intervention could have a greater impact on health care utilization, cost of care, health disparities, and quality of life [38].

In this study, we sought to assess the acceptability and feasibility of an intervention that combined the community health worker strength of family engagement and the technologic benefits of electronic medication monitoring in a high morbidity patient cohort. Prior to developing large-scale trials, we posited that first exploring acceptability and feasibility issues in this high-risk population would be critical, especially given the complex social, economic, and cultural factors. In this study, we sought to assess caregiver attitudes toward monitoring and ease of use of the devices, as well as acceptable modes and frequency of feedback, in the families of children with the highest health care utilization for asthma.

## Methods

We conducted a single-center, prospective cohort study of children with moderate to severe persistent asthma to assess the feasibility of a larger-scale intervention to improve adherence to asthma medications. The protocol for the conduct of this study was approved by The Children’s Hospital of Philadelphia Institutional Review Board.

### Study Participants

Eligible participants were children aged 3 through 16 years who received their primary care at an academic primary care clinic in West Philadelphia affiliated with The Children’s Hospital of Philadelphia, Philadelphia, PA, USA. Additional inclusion criteria were 1) enrollment in a local Medicaid managed care plan (Keystone First, Philadelphia, PA, USA), and 2) identification as a “high utilizer” of hospital and emergency services for asthma. For the purposes of this study, we defined a high utilizer as a patient whose frequency of emergency department and hospital use for asthma-related reasons was in the top 50 in the year preceding the study. Our target enrollment was 15 to 20 patients for this feasibility study. Patients were excluded from the study if they were ≥17 years of age, or had congenital heart disease, neurologic disorders, cystic fibrosis, or other chronic respiratory conditions and structural abnormalities of the upper or lower airway. Children younger than 3 years were excluded, as other wheezing illnesses may confound the diagnosis of asthma in this age group.

### Recruitment

We identified 50 potentially eligible participants using health insurance claims for the preceding year provided by the Medicaid managed care organization. An asthma navigator (a community health worker with background expertise and training in the care of children and families with asthma) contacted eligible families sequentially until 20 patients were scheduled for an initial visit. A total of 15 patients had an initial visit and 14 were enrolled in the study.

### Intervention

The study intervention included 1) motivational interviewing, 2) electronic monitoring of adherence to prescribed inhaler regimens (controller and rescue medications), and 3) outreach by the asthma navigator based on specific, predefined inhaler use criteria.

Prior to study participant enrollment, study staff members (including the asthma navigator and the study physicians) received two training sessions in motivational interviewing by experts experienced in this technique. These sessions included lectures, videos, role play of modeled behaviors, and feedback. Participant families were asked to list their three primary barriers to medication adherence at the initial study visit; these barriers were addressed using motivational interviewing techniques by the asthma navigator and, if necessary, a study physician (CK, JC, SW, or JF). Examples of such barriers were not remembering to take medications, concerns about ICS side effects, and perceptions about the severity of the child’s asthma that differed from the perceptions of the child’s clinical team. Counseling techniques for the initial visit and follow-up calls were based on strategies outlined in a review of brief motivational interviewing for asthma medication adherence by Borrelli et al [39]. To ensure fidelity of delivery, the research team developed prompts to standardize the initial portion of the telephone encounters and participated in intermittent direct observation of counseling by the asthma navigator.

**Figure 1:**
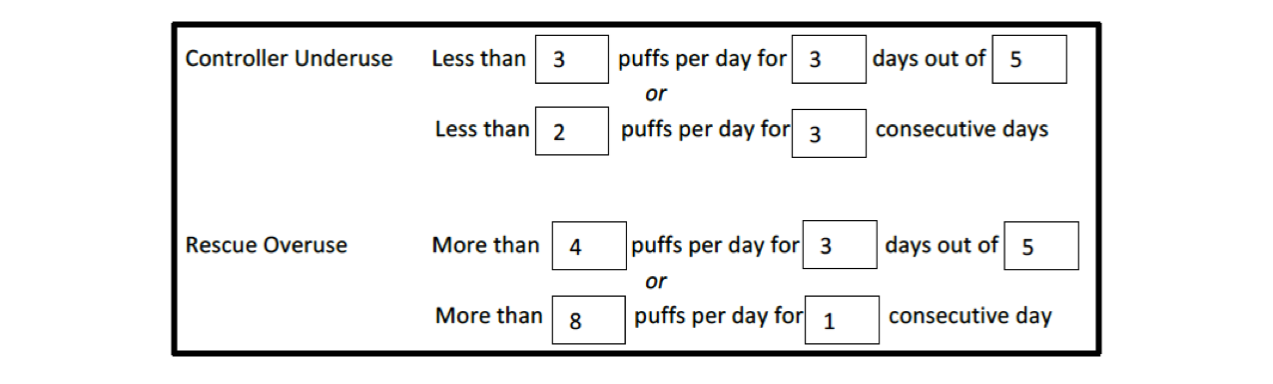
Thresholds for asthma medication alerts (based on controller schedule of 2 puffs twice a day).

Each family received SmartTouch (Adherium, Auckland, New Zealand) electronic monitoring devices for both the controller and rescue medication. SmartTouch monitors are electronic adherence monitors that can be affixed to the exterior of patients’ inhalers. Previous versions of these devices have shown both good reliability and validity in monitoring daily inhaler use [38]. Study staff affixed these devices to participants’ rescue and controller inhalers. In the case that participants did not bring their inhalers to the first study visit or had less than a month’s supply remaining, we provided participants with new rescue (albuterol) and controller inhalers (fluticasone or fluticasone-salmeterol) to ensure availability. The electronic devices transmitted inhaler usage information to a cellular modem in the home, which we also provided. Medication usage data were then uploaded to a US Food and Drug Administration-approved, Health Insurance Portability and Accountability Act-compliant website [50]. Medication usage alerts were registered for overuse of rescue medications and underuse or overuse of controller medications at predetermined frequencies (Figure 1). Families were encouraged at enrollment to take their controller medications as directed by their primary care doctor and to continue with daily and rescue asthma care as they usually would. We provided them with chargers for the devices, and instructed them on how to monitor battery life and to charge the devices when battery life was low (indicated by a red light).

The asthma navigator and a study physician (CK) actively monitored the electronic monitoring website for medication alerts. The asthma navigator contacted families after the initial study visit to ensure the study modem was connected and that the system was transmitting inhaler usage data to the monitoring website. Study staff made no subsequent contact with families for the first month to allow participants’ medication use patterns to return to their baseline. After this observation period and for the subsequent 2 months, when alerts were triggered for controller medication underuse, the asthma navigator contacted families and used motivational interviewing techniques to address suboptimal adherence. The asthma navigator monitored the site daily, except on weekends. For those with persistent daily alerts beyond 2 consecutive days, calls were attempted twice weekly. In the case of rescue medication overuse, the family was instructed to call the clinic triage nurse to determine whether a clinic appointment was needed for an asthma exacerbation. We provided basic cell phones with unlimited minutes and text messaging to families to facilitate communication with study staff during the study period.

### Outcomes

The primary outcomes for this study were acceptability and feasibility. To measure acceptance of the electronic monitoring technology, we assessed the perceived ease of use, result demonstrability, and perceived usefulness of the new technology using these subscales from the previously validated technology acceptance model instrument [40,41]. For our evaluation of feasibility, we assessed the functionality of the technology, initiation and duration of use of the electronic monitors by the caregivers of study participants, and the ability to contact study participants in response to adherence alerts.

Secondary outcomes were asthma control and daily adherence. We assessed parental perception of asthma control with the Asthma Control Test (ACT) [42]. An ACT score of ≤19 is consistent with uncontrolled asthma. ACT questionnaires were completed by participants with the asthma navigator at the initial visit and follow-up visit, and scores were recorded for comparison at the end of the study. Daily adherence was captured using the electronic monitoring devices.

### Analysis

We used descriptive statistics to characterize study participants’ demographic composition, prior health care utilization, and responses to the modified technology acceptance model questionnaire. We identified qualitative patterns of daily controller use in the month preceding asthma navigator contact with the family based on two criteria: the percentage of days in the observation window with controller actuations; and runs of consecutive days with controller actuations (see Patterns subsection of Results section). We used both paired *t* tests and Wilcoxon signed rank sum test to compare ACT scores at the start of the study with scores at the completion of the study based on the assumption of the data with and without normality. The American Thoracic Society suggests that the minimally clinically significant ACT score change is 3 points [43].

## Results

Of the 50 highest utilizers of emergency asthma care, we enrolled 14 patients for this study, of whom 6 (43%) were female. Their median age was 3.5 years (range 3–9), and all families identified their race as non-Hispanic black. Enrolled participants had a mean of 7.8 (range 5–15) combined emergency visits and hospitalizations in the preceding year, and the mean ACT score at the first visit was 15.9 (range 9–24) (Table 1).

**Table 1:** Participant characteristics (n=14).

Characteristics		Median (range) or n (%)
Age in years, median (range)		3.5 (3–9)
Female, n (%)		6 (43)
Race/ethnicity non-Hispanic black, n (%)		14 (100)
Prior emergency visits and hospitalizations, mean (range)		7.8 (5–15)
Asthma Control Test score, mean (range)		15.9 (9–24)
**Baseline adherence pattern, n (%)**		
	Sustained	4 (29)
	Periodic	5 (36)
	Lapsed	5 (36)

### Baseline Patterns

**Figure 2:**
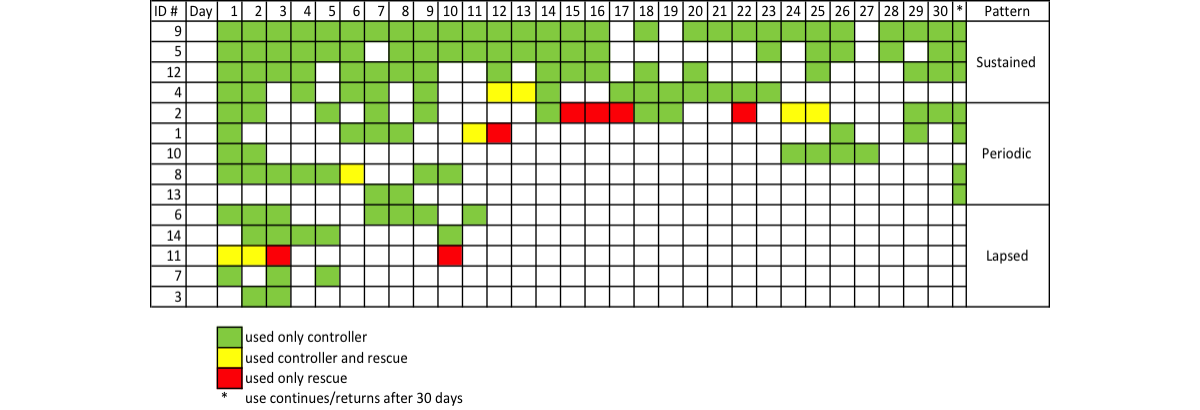
Patterns of asthma controller and rescue medication use in the first 30 days of the study. ID#: participant identification number.

We identified distinct patterns of controller use over the first 30 days of the study interval (Figure 2): 4 patients had sustained use (>50% of days with use and <7 consecutive days of missed doses), 5 patients had periodic use (<50% of days with use with >7 consecutive days with no use), and 5 patients had lapsed use (<50% of days with use and no return of use after 14 days).

### Acceptability

Of the 14 caregivers, 9 (64%) completed the modified technology acceptance model questionnaire at the final study visit. All 9 found the electronic monitoring devices to be easy to use, saw clear benefit in the devices, and would recommend the devices to others, and 5 (56%) of these caregivers felt that the device itself actually helped to improve asthma control. A total of 8 families (89%) felt that the devices were small enough to carry easily.

### Feasibility

All 14 participants initiated use of the electronic monitors; however, no modem signal was transmitted for 5 of them after a mean of 45 days. Of the 9 caregivers who completed the final study visit, 2 had lost one of the monitoring devices; we were not able to retrieve the 10 devices from the 5 caregivers who did not complete the final study visit. Thus, in total, 16 of the 28 electronic monitoring devices were returned, and 10 of them (63%) were either uncharged or no longer responding to a charge. Families who disconnected their cellular modems noted competition for use of the electrical outlets and concerns about the additional use of electricity contributing to their electricity bills. Because of these issues with data capture during the intervention window, we report adherence data for only the first 30 days of the intervention, when all participants had a transmitting modem signal.

Telephone contact was achieved with all participants (up to 2 calls per week for those with frequent adherence alerts) and ranged from short messages on their answering services to 10-minute calls to troubleshoot device issues or adherence lapses. Of the 9 caregivers who completed the final study visit, all were happy with the relationship they had with the asthma navigator, none felt that she contacted them too frequently, and 8 of them (89%) believed calls from the asthma navigator helped to avoid missing doses.

### Limited Efficacy

Though the study was not powered to detect a change in asthma control, mean ACT score improved by 2.7 points (95% CI 0–5.5, *P*=.05) over the duration of the study, just below the minimally significant ACT change threshold of 3 points.

### Loss to Follow-Up

The 5 caregivers who did not complete the final study visit were either unable to be contacted or unable to schedule and complete the final study visit following at least five attempts by the study team. Of these 5 participants, 3 were female, their median age was 3, and mean baseline ACT was 17.8 points, none of which were statistically significantly different from the characteristics of those who completed the final study visit.

## Discussion

In this study, we partnered with a local managed care Medicaid plan to assess the feasibility of an electronic monitoring intervention for high-risk inner-city minority children with asthma. To our knowledge, this is the first study to assess the acceptability and feasibility of an electronic monitoring intervention delivered by a community health worker in the highest-risk children with asthma. With respect to feasibility, our findings demonstrate several prominent challenges in the delivery and sustainability of this intervention. While all 9 of the caregivers who completed the final study visit found the technology and monitoring devices acceptable, we were not able to complete the survey for 5 families (36%). And while all of the families initiated use of the monitoring technology, half of the caregivers either unplugged the cellular modem or lost the monitoring devices, leading to difficulty in interpretation of adherence data beyond the initial observation month. Despite these challenges, we noted a trend toward improved asthma control over the study interval, and we noted some qualitatively distinct patterns of ICS use from the observation period of this study that may inform future inquiry and interventions in high-risk asthma populations.

Studies have demonstrated that electronic monitoring of ICS adherence with some form of feedback can improve medication adherence in selected populations. In a recent randomized trial of patients ages 14–65 years with poorly controlled asthma in Australia, patients who received inhaler reminders and feedback demonstrated adherence rates of 73% compared with 46% in the groups that had usual care or personalized adherence discussions [29]. A randomized trial of electronic monitoring with an audiovisual reminder in children 6–15 years old conducted in New Zealand demonstrated rates of adherence of 84% in the intervention group compared with 30% in the control [34]. An earlier review of electronic monitoring of inhaler use described the accuracy and reliability of newer electronic monitoring devices and efficacy of monitoring and feedback interventions, but highlighted feasibility concerns in vulnerable populations, including “the patient’s ability and willingness to use electronic monitoring devices” [38]. Our study offers a first glimpse of feasibility issues in a high-utilizer, inner-city minority cohort of children in the United States.

One of our primary concerns prior to launching this study was that inner-city minority families might feel that electronic monitoring interventions are overly intrusive and, thus, unacceptable. In this small study, we found no evidence to suggest this, though only 9 families (64%) completed the acceptability survey. A total of 5 (36%) families did not complete the final study visit and, though we cannot be sure of the reasons why they did not follow-up, this relatively high rate of loss to follow-up could be interpreted as their revealed lack of acceptability.

Another prominent feasibility question was whether families would initiate and maintain use of the devices. Our findings show that, while all the enrolled families effectively set up and began to use the electronic monitoring systems, there were challenges in maintaining the devices and data transmission. Several families unplugged the modem devices either temporarily or permanently, noting competition for available electrical outlets and concerns about electricity bills. Also, many of the monitoring devices that were returned had no remaining charge. These issues might have been mitigated at the onset of the study by including a power strip and communicating the negligible daily use of electricity of the cellular modem and charging devices.

With respect to outcomes, while the study was not designed to detect an improvement in asthma control, we found a statistically, but just short of clinically, significant improvement in ACT score over the course of the study for those who completed both study visits. It is important to note, however, that the study was uncontrolled; improvements in parental perception of asthma control may reflect regression to the mean or a placebo effect. We also noted distinct patterns of medication use over the first month of the study, prior to the initiating outreach for adherence alerts. Different patterns of daily medication use have been noted in one prior study of asthma in adults [44], as well as in pediatric conditions such as epilepsy [45], inflammatory bowel disease [46], and cancer [47]. While these categories have different labels in each prior study, further research should be directed at learning which potentially modifiable factors drive more sustained use patterns; this may assist families with lapsed or periodic use patterns in improving the consistency and duration of their controller medication use.

Based on the findings of this feasibility study, we offer a few considerations for future studies seeking to use electronic medication monitoring to improve the care of high-risk populations. Investigators should 1) establish regular communication with the electronic device company and gain assurance that there is robust technological support to troubleshoot technological issues that arise during the intervention, 2) consider practical issues such as competition for electrical outlets within the house and cellular or wireless coverage in the community of interest that might affect data transmission, and 3) include team members with knowledge and credibility in the community (such as community health workers) who can assist in recruitment and outreach and help elicit subtle reasons for changes in medication use or data transmission.

Our study has a few limitations. First, since this was a feasibility study, the sample size was small and restricted to one clinic population. Thus, the feasibility concerns we present may not be representative of high-risk populations in other settings. Second, the families who agreed to participate in the study (and completed the final study visit) may have been more open to and accepting of any type of intervention, inclusive of electronic monitoring, than other high utilizers or the general population. Participants’ ratings of acceptability may have been further enhanced by the receipt of a cellular phone with unlimited minutes and by social desirability bias, as this outcome was collected by a member of the study team (the asthma navigator). The feasibility of wide-scale provision of cellular phones for high-risk families would depend on the cost effectiveness of similar interventions, an outcome beyond the scope of this study. With respect to our assessment of asthma control, we assessed parental perception of asthma control using the ACT, since the median age of patients in our cohort was 3.5 years and there are no validated measures of asthma control in children younger than 4 years. More broadly, the children in our cohort were young, with the oldest child enrolled being 9 years old. Because of this, our results may not be generalizable to older children, who tend to have greater responsibility for administering their medication [48].

With respect to adherence outcomes, we used different adherence categories than an earlier adult asthma study [44] for two reasons: 1) we were not able to capture multiple inhaler actuations occurring in the same minute in the first month of the study, and 2) even with our most conservative estimate, none of our participating patients met their criteria for “compliance” (75% of prescribed doses). Lastly, our assessment of adherence beyond 1 month was substantially limited by incomplete data resulting from causes such as unplugged cellular modems and uncharged monitoring devices. Therefore, we have presented medication use data for only the first month of the study prior to the onset of these issues, but also prior to the onset of the outreach intervention.

As payers, health systems, and providers seek new approaches to improving the care of high-risk populations, new or adapted technologies for “automated hovering” offer the potential to influence the 5000 hours that patients spend outside of the reach of the health care system [49]. While some electronic ICS monitoring interventions have shown promise in other populations, our findings demonstrate some of the prominent feasibility challenges of implementation in adapting such an intervention to a vulnerable patient cohort, even despite the strength of having a community-based peer as our effector arm. The findings of this study can be used to both anticipate challenges when planning future interventions and specify areas of future inquiry germane to understanding medication use behaviors of high-risk populations.

## Abbreviations

ACT: Asthma Control Test
ICS: inhaled corticosteroids
